# Insights from Mendelian randomization and genetic correlation analyses into the relationship between endometriosis and its comorbidities

**DOI:** 10.1093/humupd/dmad009

**Published:** 2023-05-09

**Authors:** Isabelle M McGrath, Grant W Montgomery, Sally Mortlock

**Affiliations:** The Institute for Molecular Bioscience, The University of Queensland, Brisbane, QLD, Australia; The Institute for Molecular Bioscience, The University of Queensland, Brisbane, QLD, Australia; The Institute for Molecular Bioscience, The University of Queensland, Brisbane, QLD, Australia

**Keywords:** endometriosis, gynaecology, genetics, genomics, Mendelian randomization, genetic correlation, comorbidity

## Abstract

**BACKGROUND:**

Endometriosis remains a poorly understood disease, despite its high prevalence and debilitating symptoms. The overlap in symptoms and the increased risk of multiple other traits in women with endometriosis is becoming increasingly apparent through epidemiological data. Genetic studies offer a method of investigating these comorbid relationships through the assessment of causal relationships with Mendelian randomization (MR), as well as identification of shared genetic variants and genes involved across traits. This has the capacity to identify risk factors for endometriosis as well as provide insight into the aetiology of disease.

**OBJECTIVE AND RATIONALE:**

We aim to review the current literature assessing the relationship between endometriosis and other traits using genomic data, primarily through the methods of MR and genetic correlation. We critically examine the limitations of these studies in accordance with the assumptions of the utilized methods.

**SEARCH METHODS:**

The PubMed database was used to search for peer-reviewed original research articles using the terms ‘Mendelian randomization endometriosis’ and ‘“genetic correlation” endometriosis’. Additionally, a Google Scholar search using the terms ‘“endometriosis” “mendelian randomization” “genetic correlation”’ was performed. All relevant publications (n = 21) published up until 7 October 2022 were included in this review. Upon compilation of all traits with published MR and/or genetic correlation with endometriosis, additional epidemiological and genetic information on their comorbidity with endometriosis was sourced by searching for the trait in conjunction with ‘endometriosis’ on Google Scholar.

**OUTCOMES:**

The association between endometriosis and multiple pain, gynaecological, cancer, inflammatory, gastrointestinal, psychological, and anthropometric traits has been assessed using MR analysis and genetic correlation analysis. Genetic correlation analyses provide evidence that genetic factors contributing to endometriosis are shared with multiple traits: migraine, uterine fibroids, subtypes of ovarian cancer, melanoma, asthma, gastro-oesophageal reflux disease, gastritis/duodenitis, and depression, suggesting the involvement of multiple biological mechanisms in endometriosis. The assessment of causality with MR has revealed several potential causes (e.g. depression) and outcomes (e.g. ovarian cancer and uterine fibroids) of a genetic predisposition to endometriosis; however, interpretation of these results requires consideration of potential violations of the MR assumptions.

**WIDER IMPLICATIONS:**

Genomic studies have demonstrated that there is a molecular basis for the co-occurrence of endometriosis with other traits. Dissection of this overlap has identified shared genes and pathways, which provide insight into the biology of endometriosis. Thoughtful MR studies are necessary to ascertain causality of the comorbidities of endometriosis. Given the significant diagnostic delay of endometriosis of 7–11 years, determining risk factors is necessary to aid diagnosis and reduce the disease burden. Identification of traits for which endometriosis is a risk factor is important for holistic treatment and counselling of the patient. The use of genomic data to disentangle the overlap of endometriosis with other traits has provided insights into the aetiology of endometriosis.

## GRAPHICAL ABSTRACT

**Figure:**
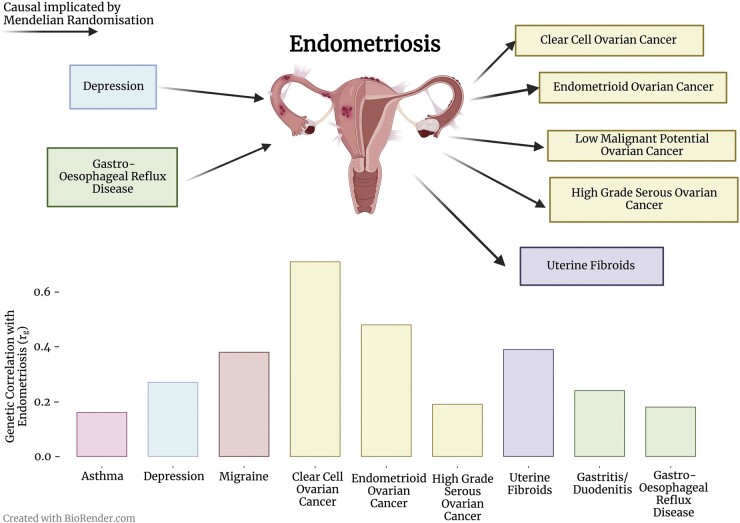
Endometriosis is associated with psychiatric (blue), gastrointestinal (green), cancer (yellow), gynaecological (purple), immune (pink), and pain (red) comorbidities through a causal mechanism and/or shared genetic background

## Introduction

### Endometriosis

Endometriosis is an oestrogen-dependent chronic inflammatory disease affecting one in nine reproductive-aged women (Rowlands *et al.*, 2021). This disease is characterized by the presence of endometrial-like tissue (stroma and glands) outside of the uterus. Unlike eutopic endometrial tissue, this ectopic endometrial tissue cannot be shed during menstruation, causing inflammation and scaring. Common disease symptoms include pelvic pain, dysmenorrhoea, dyspareunia, and infertility. Despite the penning of the term ‘endometriosis’ almost 100 years ago (Sampson, 1921, 1925, 1927), the pathogenesis remains an enigma. As such, the diagnostic method is invasive (laparoscopic surgery), and disease often recurs following treatment. An understanding of the aetiology of endometriosis is key to design preventive strategies, improve diagnosis, facilitate patient management, and develop new treatments.

One method used to uncover the biological mechanisms involved in a disease is studying the relationship between the disease and a commonly co-occurring disease. When two diseases are comorbid owing to shared molecular pathways, a comprehension of the overlap can disentangle these molecular pathways leading to disease and can also be clinically useful by improving patient management and revealing possible drug repositioning strategies. Women with endometriosis frequently experience many other conditions across multiple biological systems: pain, gynaecological, cancer, inflammatory, gastrointestinal, psychological, and altered anthropometric traits are common. This range of comorbidities suggests that multiple biological mechanisms are relevant to endometriosis. Therefore, it may be possible to utilize these conditions to provide insight into the developmental mechanisms of endometriosis. This review will detail the methods used to analyse the relationship between two traits, and then provide a discussion of the various traits implicated with endometriosis.

### Hypotheses for the causes of comorbidities

**Figure 1.**
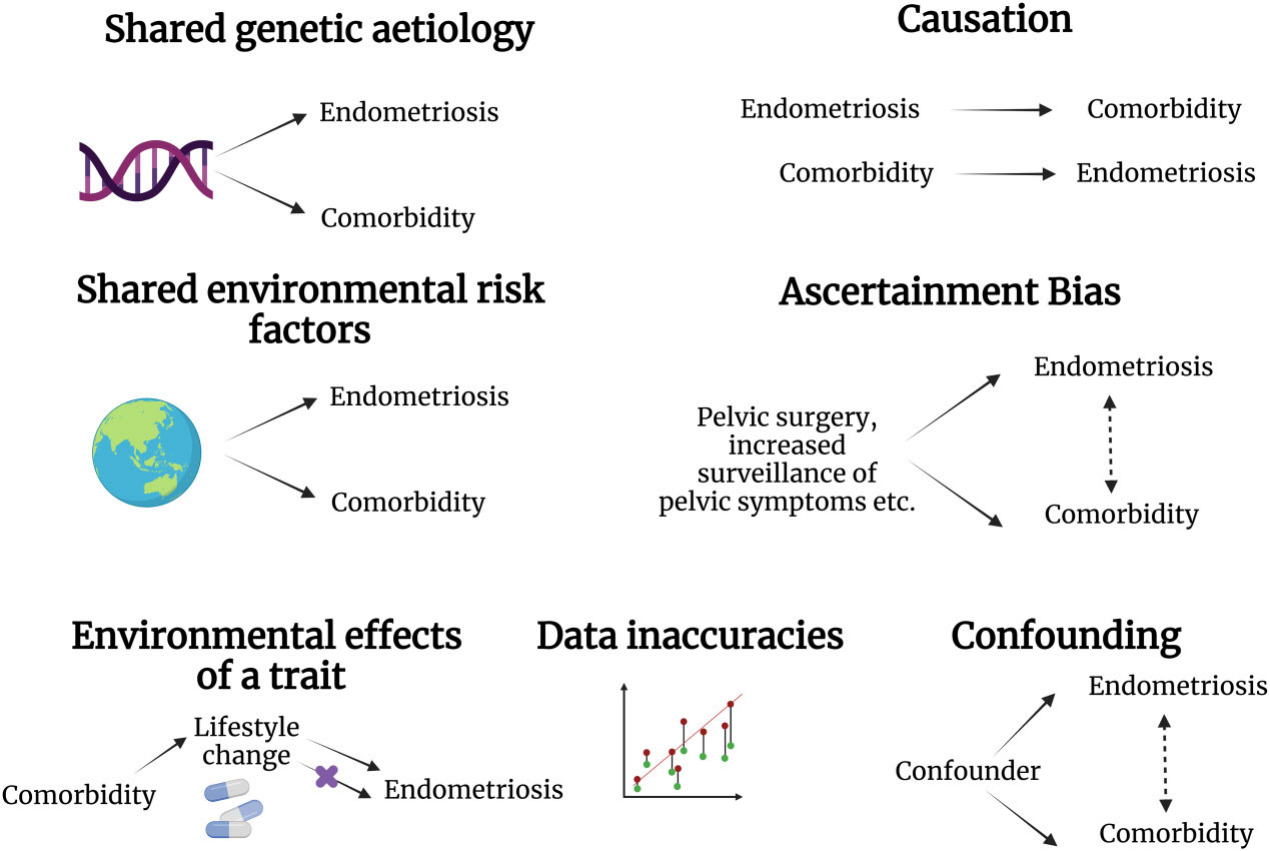
**Hypotheses for the driving factors for association of endometriosis with other traits.** Created with BioRender.com.

For this review, a comorbidity is considered a disease that occurs with increased prevalence in those with another disease; the two traits may not necessarily occur concurrently. There are several explanations when comorbidity of two traits is observed, including shared genetic aetiology (pleiotropy) or causation, shared environmental factors, ascertainment bias, lifestyle changes triggered by another trait impacting disease risk, confounding variables, and data inaccuracies (Fig. 1). First, in the case of shared genetic aetiology, two traits may share genetic risk factors, and thus likely share molecular pathways of disease. This can occur as horizontal pleiotropy where a genetic variant or gene affects two traits through independent pathways, or vertical pleiotropy/causation where one trait is an outcome of the other trait (Hemani *et al.*, 2018). These two explanations are biologically informative of disease processes. Shared environmental factors can also induce correlations between the occurrence of traits. However, environmental risk factors for endometriosis are not well characterized and as such the potential for shared environmental factors to drive endometriosis comorbidities has not been assessed. It is also possible ascertainment bias may be driving an observed association. Ascertainment bias occurs when sampling is performed in such a way that some members of the intended population have a different probability of being sampled than others. For example, undergoing any pelvic surgery (laparoscopy, hysterectomy, etc.) enables the detection of multiple gynaecological conditions, regardless of whether they were suspected. The increased risk of diagnosis of multiple other gynaecological conditions due to suspicion of one condition may amplify or create spurious associations. Lifestyle factors influenced by a diagnosis of one trait may influence the risk of another trait. Non-steroidal anti-inflammatory drugs are often prescribed to manage endometriosis symptoms. These are known to have side effects of gastrointestinal mucosal injury, which may aggravate gastrointestinal symptoms (Mahmud *et al.*, 1996). Factors such as drug use or diet may also be considered confounding variables. A confounding variable is one that is associated with both the exposure and the outcome but is not on the causal pathway between the exposure and outcome. It is important to address whether the observed comorbidity may be affected by confounding, which may be challenging when multiple factors exist concurrently, or when confounding factors are unknown. Lastly, two traits may be linked due to inaccuracies in the data. Given endometriosis cannot be easily diagnosed, many individuals with suspected endometriosis may opt not to seek a gold-standard diagnosis, and many endometriosis cases with mild symptoms may go undetected. When endometriosis studies include self-report cases, it is possible that a subset of these individuals do not have endometriosis. Similarly, control populations may contain individuals with undiagnosed disease.

### Assessing the genetic relationship between traits

Endometriosis is a complex disease, which means both genetic and environmental factors contribute to an individual’s disease liability. A recent genome-wide association study (GWAS) of 20 933 cases and 482 225 controls revealed 27 independent signals (single-nucleotide polymorphisms (SNPs)) associated with endometriosis (Rahmioglu *et al.*, 2018) and an additional six novel loci were identified in a replication analysis of 37 183 self-reported cases and 251 258 controls (Galarneau *et al.*, 2018). The heritability (*h*^2^) for endometriosis estimated via analysis of monozygotic and dizygotic twins suggests that 47–51% of the variance in liability to endometriosis is attributable to additive genetic factors (Treloar *et al.*, 1999; Saha *et al.*, 2015).

Methods utilizing the genetic architecture of traits have been favoured for investigating comorbid relationships. The act of consulting a physician increases the probability of diagnosis of other conditions, which can cause spurious associations between disorders. By analysis of independent datasets, genomic studies can be less subject to ascertainment bias for disease status. Further, additional statistical power can be leveraged by analysing all individuals with the conditions of interest, rather than limiting it to individuals with both conditions as this number is often small. Genomic data can be utilized to assess causality between traits (Smith and Ebrahim, 2003), to measure the proportion of variation shared between traits that is due to underlying genetics (Bulik-Sullivan *et al.*, 2015a,b), and to pinpoint genes and variants involved across traits (Giambartolomei *et al.*, 2014; de Leeuw *et al.*, 2015; Bakshi *et al.*, 2016; Pickrell *et al.*, 2016). The substantial genetic component of endometriosis enables investigation of its relationship to other traits through use of the genetic variants. Here the theory, assumptions and limitations of several cross-disease genomic methods are detailed.

#### Assessing causal relationships with Mendelian randomization

To determine causality, the method of Mendelian randomization (MR) can be utilized. This technique uses the genetic variants identified to be significantly associated with a trait to make causal inferences about risk factors for a disease (Smith and Ebrahim, 2003). Specifically, if an exposure trait is causal of an outcome trait, and there are multiple genetic variants causal of the exposure, the causal effect of the exposure on the outcome can be estimated by finding the ratio of the effect of the genetic variants on the outcome by the effect of the genetic variants on the exposure. This ratio is found for all variants significantly and independently associated with the exposure and combined into an overall estimate. MR can be applied to individual level genetic data or summary level data from GWASs; it is more widely used with summary data owing to greater availability.

**Figure 2.**
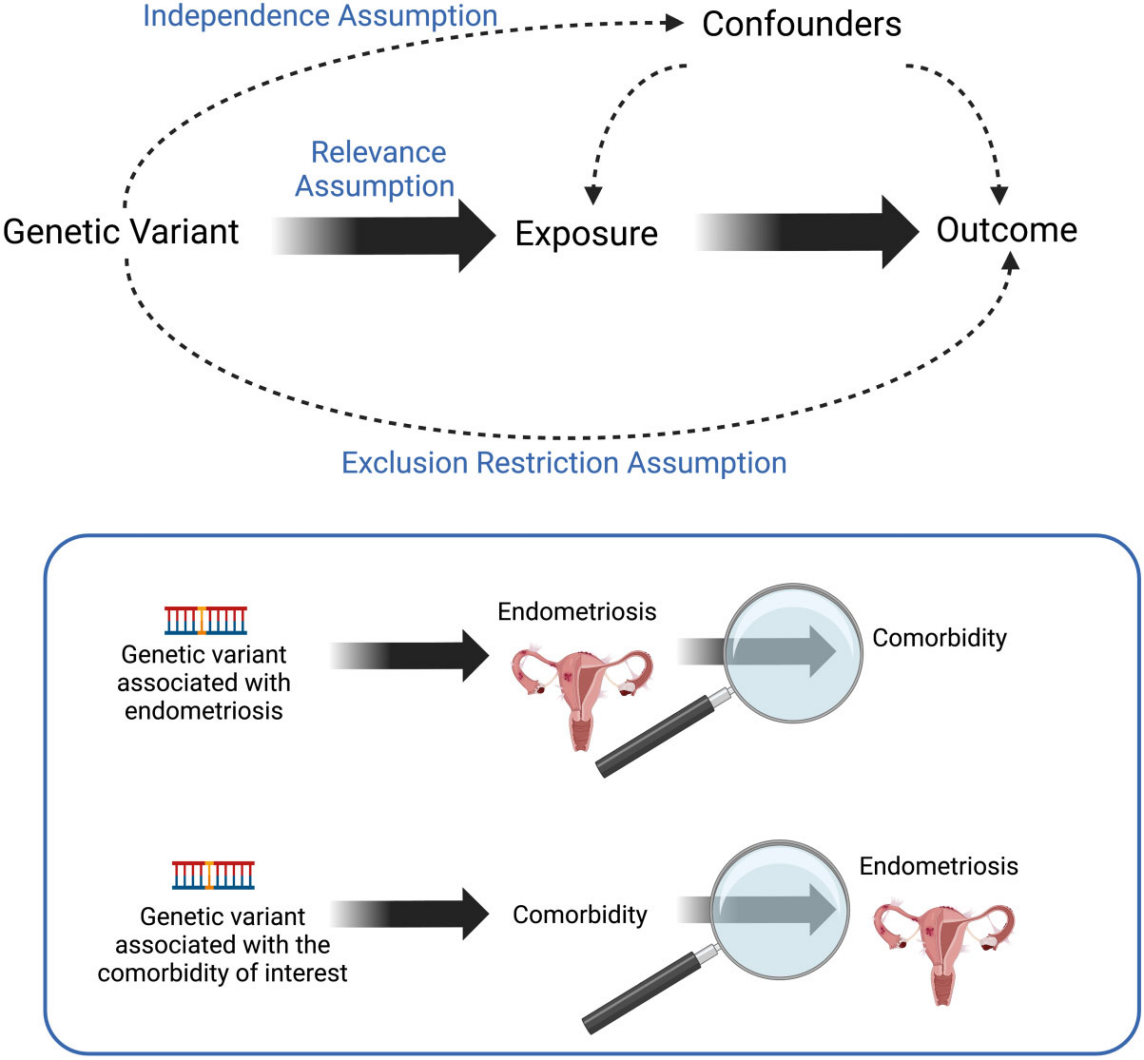
**Mendelian randomisation framework.** Mendelian randomization (MR) assesses the effect of an exposure on an outcome, by using the genetic variants associated with the exposure. There are three key assumptions of MR. *Relevance assumption:* the genetic variants must be strongly associated with the exposure variable. Therefore, only single-nucleotide polymorphisms at a genome-wide threshold of $P<5{\times 10}^{-8}$ should be utilised in the analysis. *Independence assumption:* the genetic variants must not be associated with any confounders of the exposure—outcome relationship. Confounding is challenging to detect, as it requires knowledge of all possible confounders, and high-quality datasets to assess and correct for confounding (de Leeuw *et al.*, 2022). *Exclusion restriction assumption:* the genetic variants do not affect the outcome other than through the exposure. The lower panel illustrates how MR can be applied to assess causality between endometriosis and its comorbidities. Created with BioRender.com.

MR analysis has three key assumptions that must be met for the causal estimates to be valid: relevance, independence, and exclusion restriction assumption (Fig. 2). If a variant is acting through an alternative pathway rather than directly via the exposure, for instance if it has a causative effect on another trait that also causes the outcome (a confounder), or if the variant is pleiotropic (acting directly on both the exposure and outcome), the causal effect estimated by the pleiotropic variant will likely be different to that of the other variants that satisfy the MR assumptions (de Leeuw *et al.*, 2022). Causal effect heterogeneity can be estimated, and bias can be mitigated by removing these variants (e.g. generalized summary Mendelian randomization (GSMR) (Zhu *et al.*, 2018), MR residual sum and outlier (MR-PRESSO) (Verbanck *et al.*, 2018)) or using methods that are robust to heterogenous effects. Such robust methods include methods that assume only a subset of variants are valid (e.g. weighted median: WM; Bowden *et al.*, 2016), and methods that do not require any variants to be valid, instead enacting a weaker assumption whereby heterogeneity independent from the size of the direct effect is allowed (e.g. MR-Egger; Bowden *et al.*, 2015; de Leeuw *et al.*, 2022). Conversely, the inverse variance-weighted (IVW) method does not relax any of the assumptions of MR, requiring all genetic variants to be valid instrumental variables for the estimate to be valid. When the genetic variants have a direct effect on the outcome variable, which in turn has a causal effect on the exposure variable (reverse causality), this does not manifest as heterogeneity. The true causal direction can be ascertained by performing MR in both directions and by considering which trait is known to occur first, when possible (de Leeuw *et al.*, 2022). There is substantial pleiotropy in loci implicated in gynaecological characteristics, for instance genetic variants in high linkage disequilibrium (LD) in the *FSHB* locus are linked to 11 traits, including endometriosis (McGrath *et al.*, 2021). It is also important to consider potential confounding factors that may be affecting the causal estimate. Whilst it is simple to evaluate and correct for a confounder, in practice this requires GWAS data to be available and prior evidence to consider the factor as a potential confounder (de Leeuw *et al.*, 2022). Despite these limitations in interpreting findings of MR, disentangling the pleiotropic relationships is of biological and practical interest.

#### Genetic correlation

A complementary strategy of assessing the genetic relationship between traits is genetic correlation. Unlike MR, which only uses SNPs associated with the exposure, genetic correlation uses all measured SNPs to assess correlation between effect sizes across traits. Genetic correlation is useful for traits for which the trait heritability is not well explained by the genome-wide significant SNPs, where instead the heritability is explained by many causal SNPs of small effect (Bulik-Sullivan *et al.*, 2015a,b). A genetic correlation (*r*_g_) of 0 indicates that the two traits are not genetically correlated and the genetic effects on one trait are independent of the other, whilst a correlation of 1 indicates that the genetic effects on the two traits are identical. The estimate reflects the pleiotropic effects of genes on both traits and/or the correlation between causal loci due to LD; in the instance of the latter scenario, a significant genetic correlation would not imply a true shared genetic architecture. Interpretation of the genetic correlation estimate requires the consideration of several possible relationship structures (Kraft *et al.*, 2020). Genetic correlation between traits can arise owing to: horizontal pleiotropy, whereby the two traits arise independently of each other from a shared common cause under genetic control; vertical pleiotropy, whereby one trait causes another; a shared association with an (un)observed risk factor/confounder; or multiple common causes (Kraft *et al.*, 2020). In the case of multiple common causes with opposite directions of effect, these effects may cancel out, leading to a non-significant genome-wide genetic correlation unless restricted to specific loci (Kraft *et al.*, 2020).

The estimation of genetic correlation is commonly achieved through the utilization of the readily available GWAS summary statistics. One method of genetic correlation estimation that uses GWAS summary statistics is LD score regression (Bulik-Sullivan *et al.*, 2015a,b). In this method, the correlation of effect sizes between the two traits of interest is estimated, whilst accounting for the effect of LD between variants on effect size (Bulik-Sullivan *et al.*, 2015a,b). Whilst genetic correlation using LD score regression is robust to many confounders, including environmental factors (Bulik-Sullivan *et al.*, 2015a,b; Lee *et al.*, 2018), there are a few sources of bias to which it is not robust (Kraft *et al.*, 2020). For example, LD score regression-estimated genetic correlation is susceptible to collider bias. Collider bias arises when the two traits of interest both influence a third common variable, and that variable is controlled for in the study design or analysis (van Rheenen *et al.*, 2019; Kraft *et al.*, 2020). For example, healthy individuals are more likely to participate in the UK Biobank (Fry *et al.*, 2017), which could lead to amplified genetic correlations between these ‘healthy’ traits when both samples are from the UK Biobank (Kraft *et al.*, 2020). MR is also susceptible to collider bias (Kraft *et al.*, 2020).

#### Gene-based analysis

Gene-based analyses, whereby SNPs are linked to genes, may also be conducted to enable biological interpretation of GWAS results. For a comorbidity analysis, the overlap in associated genes can be identified. Commonly used gene-based methods include fastBAT (Bakshi *et al.*, 2016) and MAGMA (de Leeuw *et al.*, 2015). The key assumption of gene-based analysis is that the gene affected by a variant is nearby the variant; however, this is not necessarily true (Sanyal *et al.*, 2012; Brænne *et al.*, 2015; Zhu *et al.*, 2016). Gene density varies throughout the genome, so assuming an even distribution of causal SNPs, a gene in a gene-poor region is more likely to be tagged by a causal variant than a gene in a gene-rich region.

#### Colocalization analysis

Several methods have been created to identify genomic regions that have causal variants shared between traits, i.e. pleiotropy. The colocalization methods pairwise GWAS (GWAS-PW) (Pickrell *et al.*, 2016) and COLOC (Giambartolomei *et al.*, 2014) use GWAS summary statistics to calculate the probability that a genomic locus contains a variant that is causal for both traits, causal for one trait, or whether there are two variants that cause each trait separately. There are limitations to this approach: in the presence of very high LD, it can be difficult to distinguish between a shared pleiotropic variant or two variants acting independently. Further, detection of shared causal variants is limited by the power in each individual study to detect causal variants. Nonetheless, these methods can be useful to elucidate the pleiotropic mechanisms underlying a comorbid relationship.

## Literature review methods

The PubMed database was used to search for peer-reviewed original research articles using the terms ‘Mendelian randomization endometriosis’ and ‘“genetic correlation” endometriosis’. Additionally, a Google Scholar search using the terms ‘“endometriosis” “Mendelian randomization” “genetic correlation”’ was performed. All relevant publications performing MR and/or genetic correlation analysis with endometriosis until 7 October 2022 are included in this review (Table 1, n = 21). Upon compilation of all traits with published MR and genetic correlation with endometriosis, additional epidemiological and genetic information on their comorbidity with endometriosis was sourced by searching for the trait in conjunction with endometriosis on Google Scholar. Traits were categorized as either pain, gynaecological, cancer, inflammatory, gastrointestinal, psychological, or anthropometric according to the major biological system or process involved.

**Table 1.** **Publications detailing Mendelian randomization and genetic correlation analyses with endometriosis**.

Trait category	Traits	Methods	Ancestry	Publication
**Pain**	Migraine	Genetic correlation and Mendelian randomization	Endometriosis: 93% European, 7% Japanese Migraine: European	Adewuyi *et al.* (2020)
		Genetic correlation	Australian	Nyholt *et al.* (2009)
**Gynaecological conditions**	Age at menarche, age at menopause, AMH levels, reproductive hormone levels and length of menstrual cycle	Mendelian randomization	Endometriosis: Finnish Age at menarche, age at menopause, AMH levels, reproductive hormone levels and length of menstrual cycle: European	Garitazelaia *et al.* (2021)
	Uterine fibroids	Genetic correlation and Mendelian randomization	Endometriosis: European Uterine fibroids: European	Gallagher *et al.* (2019)
	Age at first birth and age at first sexual intercourse	Genetic correlation	Endometriosis: European Age at first birth and age at first sexual intercourse: European	Mills *et al.* (2021)
**Cancer**	Epithelial ovarian cancer and epithelial ovarian cancer subtypes	Genetic correlation and Mendelian randomization	Endometriosis: European Epithelial ovarian cancer and epithelial ovarian cancer subtypes: European	Mortlock *et al.* (2022)
		Mendelian randomization	Endometriosis: European Epithelial ovarian cancer and epithelial ovarian cancer subtypes: European	Lu *et al.* (2015)
		Mendelian randomization	Endometriosis: 93% European, 7% Japanese Epithelial ovarian cancer and Epithelial ovarian cancer subtypes: European	Yarmolinsky *et al.* (2019)
	Ovarian cancer, ovarian cancer subtypes, breast cancer, endometrial cancer	Mendelian randomization	Endometriosis: 93% European, 7% Japanese Ovarian cancer, ovarian cancer subtypes, breast cancer, endometrial cancer: European	Rueda-Martinez *et al.* (2021)
	Melanoma	Genetic correlation and Mendelian randomization	Endometriosis: European Melanoma: European	Yang *et al.* (2021)
	Endometrial cancer	Genetic correlation	Endometriosis: Australian and UK Endometrial cancer: Australian and UK	Painter *et al.* (2018)
		Genetic correlation and Mendelian randomization	Endometriosis: 0.5% Japanese, 99.5% European Endometrial cancer: European	Kho *et al.* (2021)
**Inflammatory**	Asthma	Genetic correlation and Mendelian randomization	Endometriosis: 93% European, 7% Japanese Asthma: European	Adewuyi *et al.* (2022)
	Coeliac disease, systemic lupus erythematosus, Sjögren’s syndrome, rheumatoid arthritis, and multiple sclerosis	Mendelian randomization	Endometriosis: Finnish Coeliac disease, systemic lupus erythematosus, Sjögren’s syndrome, rheumatoid arthritis, and multiple sclerosis: European	Garitazelaia *et al.* (2021)
**Gastrointestinal**	Gastritis/duodenitis, gastro-oesophageal reflux disease	Genetic correlation and Mendelian randomization	Endometriosis: European (93%), Japanese (7%) Gastritis/duodenitis, gastro-oesophageal reflux disease: European	Adewuyi *et al.* (2021)
**Psychological**	Depression	Genetic correlation and Mendelian randomization	Endometriosis: European (93%), Japanese (7%) Depression: European	Adewuyi *et al.* (2021)
**Anthropometric**	Weight, BMI, height, WHR	Mendelian randomization	Endometriosis: Finnish Weight, BMI, height, waist-to-hip ratio (WHR): European	Garitazelaia *et al.* (2021)
	Birth weight	Mendelian randomization	Endometriosis: Finnish Birth weight: European	He *et al.* (2022)
	BMI, WHR and WHRadjBMI, each adjusted for leptin, fasting insulin and insulin sensitivity separately, as well as unadjusted Leptin, fasting insulin, and insulin sensitivity each adjusted for BMI, WHR and WHRadjBMI separately, as well as unadjusted visceral adipose tissue mass	Mendelian randomization	Endometriosis: Australia and UK, UKB and FinnGen BMI, WHR, WHRadjBMI: European. VAT mass: UKB individuals of British descent. Leptin, fasting insulin, insulin sensitivity: European.	Venkatesh *et al.* (2022)
	Adulthood and childhood BMI	Mendelian randomization	Endometriosis: UKB Adulthood and childhood BMI: European	Yan *et al.* (2022)
	BMI	Mendelian randomization	Endometriosis: Japanese BMI: Japanese	Masuda *et al.* (2020)

AMH, anti-Müllerian Hormone; UKB, UK Biobank; WHR, waist-to-hip ratio.

## Comorbidities of endometriosis

Many conditions have been identified as comorbid with endometriosis; however, their link with endometriosis is not well understood. In addition to using comorbidities to understand disease pathogenesis and heterogeneity, knowledge of the comorbidities has the potential to inform clinical predictors of disease and disease management strategies. Understanding the comorbidities of endometriosis may also help explain shared symptomology and can aid clinical decisions of screening for other conditions. Identifying the biological network of traits related to endometriosis is vital for addressing all the patient’s needs. Here, the genetic evidence for a link between endometriosis and pain, gynaecological, cancer, inflammatory, gastrointestinal, psychological, and anthropometric traits is critically examined, with the limitations of the associated methodology and epidemiological evidence also considered.

### Pain and endometriosis

Pain related to the female gynaecological organs—dysmenorrhoea/abdominal pain, dyspareunia, and chronic pelvic pain—is considered symptoms of endometriosis and is experienced by 65%, 56%, and 26% of cases, respectively (Signorile *et al.*, 2022). However, the severity of pain does not correlate with disease severity or lesion location (Schliep *et al.*, 2015; Vermeulen *et al.*, 2021), suggesting that the pain may arise through pathways beyond the lesion characteristics. The genetic relationship of endometriosis with the most reported pain symptoms—dysmenorrhoea, chronic pelvic pain and dyspareunia—has not been investigated, owing to a lack of well-powered GWASs. Pain in endometriosis cases extends beyond the pelvis: data from the Skåne Healthcare Register suggest that endometriosis is a risk factor for subsequent fibromyalgia (incident rate ratios (IRR) = 2.83, 95% CI: 1.96–4.08) and chronic widespread pain diagnosis (IRR = 5.02, 95% CI: 3.10–8.13) (Larrosa Pardo *et al.*, 2019). An Australian demographic questionnaire indicated low back pain, headache/migraine, body aches and fibromyalgia are enriched in endometriosis cases (Evans *et al.*, 2021). The risk of migraine is also increased in women with endometriosis (odds ratio (OR) = 1.70, 95% CI: 1.59–1.82) (Yang *et al.*, 2012) and in adolescents with endometriosis (OR = 4.77, 95% CI: 2.53–9.02) (Miller *et al.*, 2018). The pathways underlying the overlap between endometriosis and bodily pain have not been extensively investigated. Experimental studies suggest endometriosis patients have a decreased pain tolerance compared to individuals without an endometriosis diagnosis (van Aken *et al.*, 2018; Vuontisjarvi *et al.*, 2018). A recent review suggests that inflammation, neurogenic inflammation, neuroangiogenesis, peripheral sensitization, and central sensitization may play a role in endometriosis-related pain (Maddern *et al.*, 2020).

**Figure 3.**
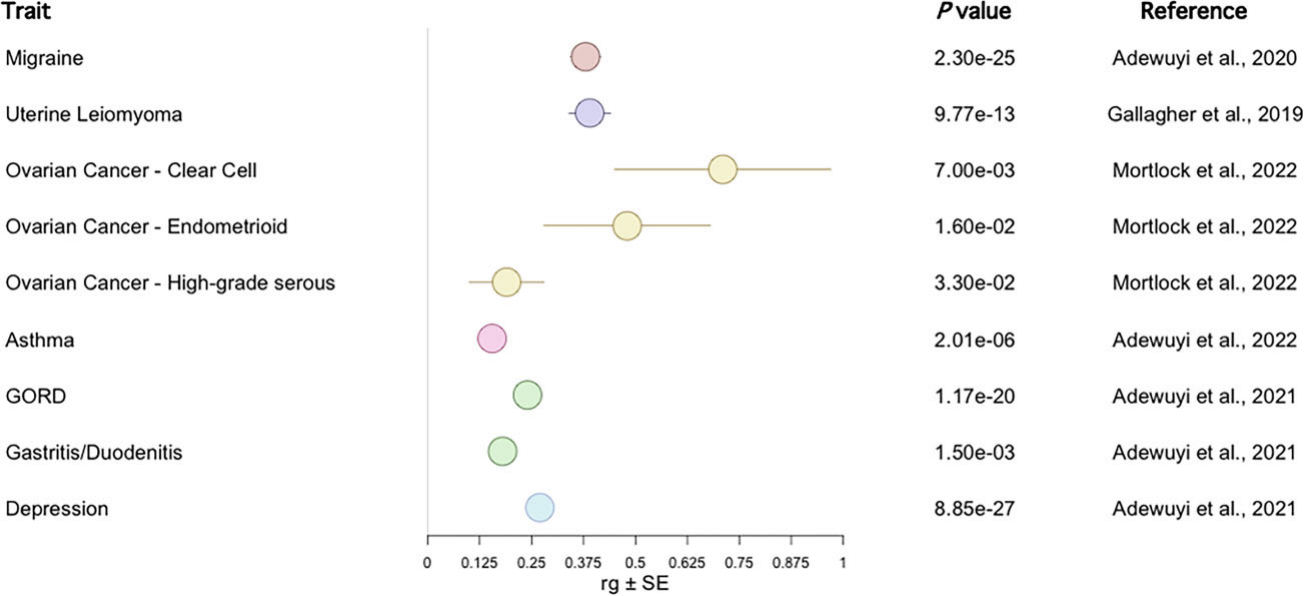
**Published genetic correlations with endometriosis.** Genetic correlations (*r*_g_) ± SE reported as significant in the original publication are shown. Where two studies have reported the genetic correlation with endometriosis for the same trait, the study utilizing the greatest powered genome-wide association study summary statistics was considered for inclusion. Red: pain traits, purple: gynaecological, yellow: cancer traits, pink: immune, green: gastrointestinal, blue: psychiatric. GORD: gastro-oesophageal reflux disease. Note an SE for GORD and gastritis/duodenitis was not provided in the original publication.

The genetic overlap of migraine with endometriosis has been investigated by Adewuyi *et al.* (2020). Migraine was reported as significantly genetically correlated with endometriosis (genetic correlation (*r*_g_) = 0.38) (Fig. 3) (Adewuyi *et al.*, 2020). A genetic correlation has also been reported with individual-level data (Nyholt *et al.*, 2009). MR analysis using SNPs associated at a genome-wide significance threshold (*P *<* *5 × 10^−8^) found no evidence for a causal relationship of endometriosis on migraine with the IVW, MR-Egger and WM methods, or vice versa (Adewuyi *et al.*, 2020). The MR-Egger pleiotropy test was not significant, indicating that there was no average pleiotropic effect across variants. The heterogeneity tests, measures of the variability in the estimated causative effect of the exposure on the outcome across variants, did not provide convincing evidence of such variability. In single variant MR analysis, one endometriosis-associated variant, rs74485684, showed evidence for a risk-increasing effect on migraine (Adewuyi *et al.*, 2020). This variant is also associated with menstrual cycle length and excessive, frequent and irregular menstruation: potential risk factors for both endometriosis and migraine. Exclusion of this variant from the MR analysis did not alter the results (Adewuyi *et al.*, 2020). This study utilized endometriosis summary statistics published in Sapkota *et al.* (2017), which were derived from individuals of European (93%) and Japanese (7%) ancestry, whilst the migraine summary statistics and the LD reference panel were 100% European. This may cause bias for MR and genetic correlation (Bulik-Sullivan *et al.*, 2015a,b) as LD scores differ between populations, although bias is expected to be small given the small proportion of non-Europeans. A gene-based analysis revealed 17 genes are shared between endometriosis and migraine (*P*_gene_ < 0.01). Functional enrichment analysis revealed three biological clusters involved in both diseases: mitogen-activated protein kinase (MAPK) signalling pathway, regulation of kappa-light-chain-enhancer of activated B cells signalling, and tumour necrosis factor alpha signalling pathway. These clusters suggest sex hormones, inflammation, and protein adhesion and phosphorylation are important for both disorders (Adewuyi *et al.*, 2020). The authors recommend clinicians should be wary of migraine as a comorbidity in endometriosis cases (Adewuyi *et al.*, 2020). This is particularly important given combined hormonal contraceptives are often prescribed to manage the symptoms of endometriosis but can exacerbate migraines (Granella *et al.*, 1993; Aegidius *et al.*, 2006). However, recent evidence suggests certain contraceptives can reduce migraine attacks (Allais *et al.*, 2017).

### Gynaecological conditions and endometriosis

#### Menstrual cycle related traits

Endometriosis lesions contain cells that closely resemble those in eutopic endometrial tissue. This, combined with the commonly accepted theory of retrograde menstruation for the origin of endometriotic cells, and epidemiological data, suggests a potential influence of menstrual characteristics on disease. Individuals with endometriosis have been observed to have a younger age of menarche (Missmer *et al.*, 2004; Treloar *et al.*, 2010; Nnoaham *et al.*, 2012) and shorter menstrual cycle length (Wei *et al.*, 2016). An earlier age of menarche and more frequent cycles implies a greater exposure to menstruation, supporting Sampson’s theory of retrograde menstruation for endometriosis development. This evidence for the retrograde menstruation theory is further supported by the observation of common somatic mutations between eutopic endometrium and the ectopic endometriosis lesions (Suda *et al.*, 2018, 2020). The impact of ovarian endometriomas on age of menopause onset has also been debated, with hypothesized mechanisms of effect being through the surgical treatment of these lesions reducing ovarian reserve, or through an effect exerted by the lesion itself on the ovary (Yilmaz Hanege *et al.*, 2019). Individuals with endometriosis have also reported having a higher menstrual flow than controls (Vercellini *et al.*, 1997). A review of medical records of 5540 cases of endometriosis and 21 239 controls indicated cases were more likely to experience heavy menstrual bleeding (OR = 5.0, 95% CI: 4.6–5.5) (Ballard *et al.*, 2008).

Age of menarche, age of menopause, anti-Müllerian hormone (AMH) levels, reproductive hormone levels and length of menstrual cycle have been assessed as potential causes of endometriosis using MR analysis, using the WM, MR-Egger (MRE), and IVW models (Garitazelaia *et al.*, 2021). Preliminary evidence for a role for earlier age of menarche (OR_WM_ = 0.63, *P*_WM_ = 0.027), lower AMH levels (OR_WM_ = 0.66, *P*_WM_ = 0.008; OR_IVW_ = 0.68, *P*_IVW_ = 0.001), and shorter length of menstrual cycle (OR_WM_ = 0.38, *P*_WM_ = 0.003; OR_MRE_ = 0.10, *P*_MRE_ = 0.022; OR_IVW_ = 0.37, *P*_IVW_ = 0.013) was presented (Garitazelaia *et al.*, 2021). However, further analysis is needed to assess the presence of heterogeneity and pleiotropy, and to assess whether the result is consistent in a highly powered endometriosis dataset. Further, given the timing of some of these traits (e.g. menopause) typically occurring after endometriosis diagnosis, it would be particularly apt to assess endometriosis as the exposure variable. Biologically, understanding how endometriosis is linked to menstrual characteristics will aid in disentangling the role of hormonal regulation in endometriosis. This requires careful partitioning of pleiotropic effects to firstly understand the action of particular genes in endometriosis, but also to enable consideration of causality. Clinically, understanding the causal relationships with reproductive-lifespan related traits may aid diagnosis when considering menstrual characteristics as risk factors and may be personally important to the health of the patient.

#### Uterine fibroids

**Figure 4.**
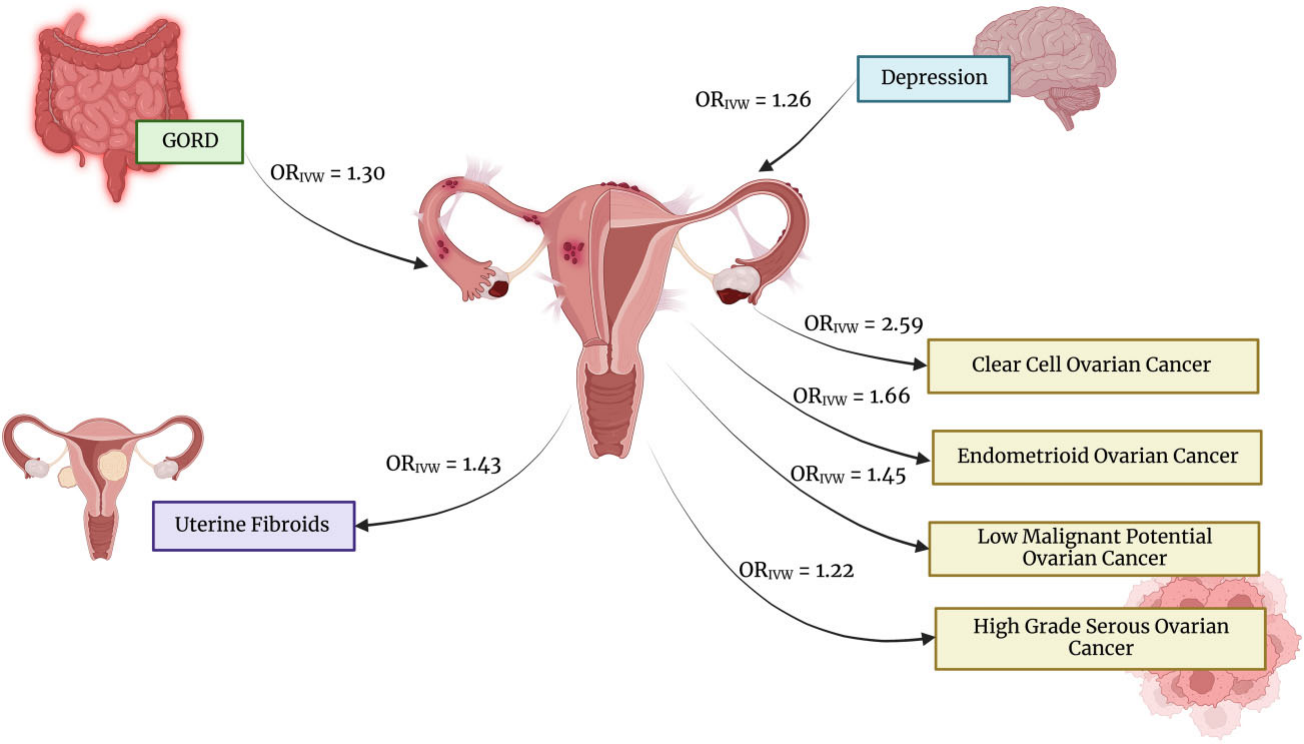
**Causal relationships with endometriosis, determined using Mendelian randomization.** The inverse variance-weighted (IVW) odds ratio (OR) reported as significant in the original publication are shown. Only relationships determined from the most well-powered and well-designed Mendelian randomization studies are depicted. The arrows indicate the direction of the relationship with gastro-oesophageal reflux disease (GORD), depression (Adewuyi *et al.*, 2021), uterine fibroids (Gallagher *et al.*, 2019) and epithelial ovarian cancer histotypes (Mortlock *et al.*, 2022). Created with BioRender.com.

Uterine fibroids, also referred to as uterine leiomyomas, are noncancerous growths of muscle on the walls of the uterus. An endometriosis diagnosis doubles the risk for uterine fibroids (multivariable-adjusted summary relative risk = 2.17, 95% CI: 1.48–3.19) and a large positive genetic correlation between the two diseases has been reported (*r*_g_ = 0.39, Fig. 3) (Gallagher *et al.*, 2019). MR suggests that genetic liability to endometriosis is causative of uterine fibroids (beta_IVW_ = 0.36, Fig. 4), which agrees with the average age of onset being younger for endometriosis than uterine fibroids (Gallagher *et al.*, 2019). Whilst significant heterogeneity was detected, there is evidence to believe the result is reasonable: in a minimal set of SNPs without heterogeneity the causal relationship remained significant (although attenuated: beta_IVW_ = 0.12); in leave-one-out sensitivity testing the result could not be attributed to an individual SNP; and the inverse relationship was not significant (Gallagher *et al.*, 2019). Four loci are significant for both traits in independent GWASs, which are linked to four genes involved in oestrogen or progesterone signalling (*WNT4/CDC42*, *GREB1*, *ESR1*, and *FSHB*) (Gallagher *et al.*, 2019). The genetic data supports the strong pathophysiological tie between the two diseases. Somatic mutation data to track the clonal lineage in an individual with concurrent endometriosis and uterine fibroids may provide further insights into the underlying biology of both conditions, and further explain the link between the diseases.

#### Fertility

Whilst 20–50% of women with infertility have endometriosis (Mahmood and Templeton, 1991; Meuleman *et al.*, 2009; Petre *et al.*, 2021), the link is not well understood (Filip *et al.*, 2020). Physiologically, endometriosis-associated infertility has been suggested to arise from adhesions, which are common in endometriosis due to the inflammatory response, as they can cause anatomic distortion of the reproductive organs (Somigliana *et al.*, 2012). Few studies have attempted to characterize the shared aetiology using genomics. Fertility is a complicated and heterogenous phenotype to measure, therefore other phenotypes can be used as proxies for fertility. To our knowledge, this has been limited to assessing the genetic correlation between age at first birth and age at first sexual intercourse in females with endometriosis and severe endometriosis (Mills *et al.*, 2021). Although the estimates were non-significant, they had large CIs owing to small sample sizes (Mills *et al.*, 2021). Further, given that these proxy traits are highly multi-factorial, these results do not negate a genetic tie. Other proxies should be considered (e.g. number of live births), in addition to comparing the genomic profile between endometriosis cases with and without fertility issues. Considering the strong epidemiological link between endometriosis and fertility and the impact of fertility issues on an individual, further genomic studies are warranted to better understand the overlap.

### Cancer and endometriosis

#### Ovarian cancer

Although endometriosis lesions are considered benign, they share several properties with cancer, including invasion, adhesion, and proliferation. Increasing evidence implicates a link between the endometrioma lesion site and epithelial ovarian cancer (EOC) (Buis *et al.*, 2013; Saavalainen *et al.*, 2018; Capmas *et al.*, 2021). Such a link was first proposed in 1925 by Sampson, who suggested a theory of malignant transformation of endometriosis (Sampson, 1925). A recent large epidemiological study revealed ovarian cancer was reported in 0.34% of women without endometriosis and 0.71% of women with endometriosis, corresponding to an adjusted OR (95% CI) of 2.61 (2.41–2.82) (Capmas *et al.*, 2021). The EOC subtypes endometrioid carcinoma (EC) and clear cell carcinoma (CCC), each accounting for ∼10% of EOC, have been consistently linked with endometriosis (Pearce *et al.*, 2012; Saavalainen *et al.*, 2018; Mortlock *et al.*, 2022). Associations with low grade serous have also been reported (Pearce *et al.*, 2012). Interestingly, patients with EOC with a history of endometriosis experience longer survival (Hermens *et al.*, 2021), lower grade, and earlier stage disease (Kim *et al.*, 2014) than those without a history of endometriosis. It has been debated whether ovarian cancer in endometriosis cases represents a distinct entity of ovarian cancer (Eržen *et al.*, 2001; Munksgaard and Blaakaer, 2011; Bounous *et al.*, 2016). Women with endometriosis are suspected to have increased probability of early ovarian cancer detection, owing to greater awareness of physiological changes and frequent gynaecological ultrasounds. Although these studies adjusted for relevant confounders, such as ovarian cancer stage (Kim *et al.*, 2014; Hermens *et al.*, 2021), within-stage differences may still be present (Hermens *et al.*, 2021). Nevertheless, this is important to consider when acknowledging the 5-year survival rate of ovarian cancer is ∼47% (Torre *et al.*, 2018; Australian Institute of Health and Welfare, 2021). A significant genetic correlation has been reported for endometriosis with CCC (*r*_g_ = 0.71), EC (*r*_g_ = 0.48), and high-grade serous ovarian cancer (*r*_g_ = 0.19) (Fig. 3), but not for low malignant potential serous ovarian cancer or mucinous ovarian cancer (Mortlock *et al.*, 2022). Similar genetic correlation results were reported in a less-powered analysis (Lu *et al.*, 2015). MR using the IVW method confirmed a causal relationship for endometriosis on CCC (OR_IVW_ = 2.59), EC (OR_IVW_ = 1.66), and high-grade serous ovarian cancer (OR_IVW_ = 1.22), in addition to low malignant potential ovarian cancer (OR_IVW_ = 1.45) (Fig. 4) (Mortlock *et al.*, 2022). These relationships were supported by other MR methods. The MR-PRESSO method (Verbanck *et al.*, 2018) indicated evidence for pleiotropy for high-grade serous ovarian cancer, yet removal of the heterogenous SNPs did not affect the estimate (Mortlock *et al.*, 2022). The MR-Egger intercept test also did not find evidence of overall pleiotropy (Mortlock *et al.*, 2022). These relationships were unidirectional—i.e. genetic liability to ovarian cancer did not exert a causative effect on endometriosis (Mortlock *et al.*, 2022). Similar results were previously published for overall ovarian cancer, the EC and CCC subtypes (Yarmolinsky *et al.*, 2019; Rueda-Martinez *et al.*, 2021), low malignant potential tumours and high grade serous ovarian cancer (Yarmolinsky *et al.*, 2019). Epidemiological evidence suggests the increased risk of ovarian cancer is restricted to the endometrioma lesion location (Saavalainen *et al.*, 2018); however, the MR and genetic correlation studies have not taken this into account as lesion location specific genetic risk variants have not yet been identified. A causal role for endometriosis on ovarian cancer implicated by MR studies is supported by somatic mutation data from an individual with concurrent endometrioma and CCC (Suda *et al.*, 2020). In this individual, a cell clonal lineage was observed progressing from the eutopic endometrium, to the endometrioma, to the clear cell ovarian cancer (Suda *et al.*, 2020). A causal link between endometriosis and ovarian cancer has important implications for screening and promotes the need for further investigations into ways of disrupting this link for cancer prevention.

#### Melanoma

Epidemiological analysis suggests endometriosis is associated with multiple pigmentary traits, family history of melanoma (relative risk = 1.13, 95% CI: 1.01–1.26) (Kvaskoff *et al.*, 2014) and melanoma (hazards ratio (HR) =1.64, 95% CI 1.15–2.35) (Farland *et al.*, 2017). A nominally significant genetic correlation has been found between endometriosis and melanoma in a female-only melanoma GWAS (*r*_g_ = 0.14). Interestingly, MR indicated a small causative effect of genetic liability to melanoma in females on endometriosis (beta_IVW_ = 0.06), but not for the reverse relationship (Yang *et al.*, 2021). This is surprising given endometriosis typically occurs earlier in life than melanoma. There was significant pleiotropy, indicating some SNPs may be influencing endometriosis through alternative pathways to melanoma (Yang *et al.*, 2021). GSMR, a tool that estimates a causal effect after removal of SNPs showing evidence of directional pleiotropy, also identified a small significant risk effect on endometriosis (beta_GSMR_ = 0.05) only for the female melanoma dataset (Yang *et al.*, 2021). Analysis of genomic loci influencing both traits with GWAS-PW suggested that no region contained a single causal variant that influences both traits, instead 27 regions showed evidence for distinct causal variants influencing the two traits (Yang *et al.*, 2021). Of the 27, 2 regions, chr9:20464018-22205246 around *CDKN2A* and chr6:19207758-21683982 around *CDKAL1*, showed the strongest evidence of two distinct causal variants (Yang *et al.*, 2021). The connection between genetic liability to endometriosis and melanoma is difficult to explain physiologically, although Yang *et al.* (2021) notes that melanocytes express oestrogen receptors (ER) and oestrogen is believed to play a role in melanoma (Mori *et al.*, 2006; Schmidt *et al.*, 2006; Koomen *et al.*, 2009; Marzagalli *et al.*, 2015; Yang *et al.*, 2021). Given the relatively small causal effect and genetic correlation between endometriosis and melanoma, any clinical utility of the relationship is likely limited and larger sample sizes would be needed to validate any genetic relationship and shared molecular pathways.

#### Endometrial cancer

Given endometriosis is hypothesized to be derived from endometrial tissue, the relationship between endometrial cancer and endometriosis has also been investigated. Epidemiological studies have shown conflicting evidence: a Danish nation-wide cohort study of 45 790 women with endometriosis identified an increased risk of endometrial cancer (standardized incidence ratio (SIR) >1 year after endometriosis diagnosis 1.43, 95% CI: 1.13–1.79); however, this study did not adjust for confounders such as parity or oral contraceptive use (Mogensen *et al.*, 2016). Meanwhile, a study of 97 109 US nurses found no association with endometrial cancer, both with and without adjustment for potential confounders (Poole *et al.*, 2017). The participants in the endometrial cancer analysis by Poole *et al.* (2017) are relatively young (∼45 years of age), which may have hindered the ability to detect an association given that Mogensen *et al.* (2016) reported a median age of endometrial cancer diagnosis of 59 years. Although one study reports a significant genetic correlation between endometriosis and endometrial cancer (*r*_g_ = 0.23) (Painter *et al.*, 2018), an updated estimate utilizing GWAS summary statistics with a large sample size did not find a significant genetic correlation (Kho *et al.*, 2021). Instead, seven shared candidate susceptibility genes in four regions were reported, of which one region (17q21.32) showed evidence of a shared genetic risk signal (Kho *et al.*, 2021). Another study suggested the relationship between endometriosis and endometrial cancer is likely explained by horizontal pleiotropy rather than causality, owing to a significant MR-Egger intercept test (Rueda-Martinez *et al.*, 2021). This result was updated by Kho *et al.* (2021) who used better-powered endometriosis GWAS summary statistics. Kho *et al.* (2021) did not find evidence for a causative effect of endometriosis on endometrial cancer, which was robust across multiple MR methods, although overall heterogeneity and pleiotropy tests were not conducted. Therefore, there is no compelling evidence of a causal effect of endometriosis on endometrial cancer.

#### Breast cancer

There is inconsistent evidence that individuals with endometriosis have an increased risk of breast cancer. The epidemiological studies investigating this relationship variably reported both a risk increasing and risk decreasing effect of endometriosis on breast cancer. The inconsistency could be caused by small sample sizes, uncontrolled confounders, or poor study design (Pontikaki *et al.*, 2016). A more recent, well-powered study on the Danish National Patient Register found an increased risk of breast cancer only among women who were diagnosed with endometriosis at ≥50 years of age (SIR 1.27, 95% CI: 1.12–1.42) (Mogensen *et al.*, 2016). Another large study based on the US Nurses’ Health Study II also failed to find an association of endometriosis with overall breast cancer but did find that endometriosis was associated with increased risk of ER+/PR– breast tumours (adjusted HR = 1.9, 95% CI: 1.44–2.50) (Farland *et al.*, 2016). MR analyses did not implicate a causative effect of endometriosis on breast cancer, nor was there evidence of overall horizontal pleiotropy; however significant heterogeneity was detected by IVW and MR-Egger methods (Rueda-Martinez *et al.*, 2021). Given epidemiological evidence suggests that endometriosis is a risk factor only for variable subsets of breast cancer, the link between endometriosis and breast cancer requires further investigation.

### Inflammatory traits and endometriosis

Endometriosis is considered a chronic inflammatory disease; therefore, it is unsurprising many other immune-related conditions are comorbid with endometriosis. The severity of endometriosis has been linked with autoimmune disease: concomitant autoimmunity was a significant predictor of stage IV endometriosis (OR = 2.54, 95% CI = 1.57–4.10) (Vanni *et al.*, 2021). A systematic review of epidemiological evidence for an association of multiple autoimmune diseases with endometriosis noted most studies suffered from bias, small sample sizes, and wide CIs (Shigesi *et al.*, 2019). A 2021 cross-sectional study of 551 surgically-diagnosed cases and 652 controls found individuals with asthma (OR = 1.35, 95% CI = 0.97–1.88), chronic fatigue syndrome and/or fibromyalgia (OR = 5.81, 95% CI = 1.89–17.9), mononucleosis (OR = 1.75, 95% CI = 1.14–2.68), and allergy (OR = 1.76, 95% CI = 1.32–2.36) have an increased risk of also having endometriosis (Shafrir *et al.*, 2021). Utilization of a large Japanese health insurance claims database of 30 516 cases of endometriosis and 120 976 controls found a significant association of type 1 allergy and endometriosis (IRR = 1.10, 95% CI: 1.06–1.13) and between rheumatoid arthritis and endometriosis (IRR = 1.31, 95% CI: 1.05–1.64), whilst systemic lupus erythematosus was not associated with endometriosis (Yoshii *et al.*, 2021). Garitazelaia *et al.* (2021) conducted MR analyses to evaluate the causative effect of coeliac disease, systemic lupus erythematosus, Sjögren’s syndrome, rheumatoid arthritis, and multiple sclerosis on endometriosis. The authors reported a preventative effect of multiple sclerosis on endometriosis (OR = 0.68) using the WM method; however, this was not supported by the MR-Egger or IVW methods, nor were tests conducted for heterogeneity or horizontal pleiotropy, and the endometriosis dataset suffered from limited power (Garitazelaia *et al.*, 2021).

The genetic relationship between endometriosis and asthma was extensively investigated by Adewuyi *et al.* (2022). A significant genetic correlation was reported (*r*_g_ = 0.16, Fig. 3), although MR analysis failed to identify a causative relationship in either direction (Adewuyi *et al.*, 2022). Cross-disorder meta-analysis identified 26 independent SNPs as shared between disorders, a subset of which has not previously been reported as associated with either disease (Adewuyi *et al.*, 2022). GWAS-PW identified 37 regions with evidence of a shared causal variant between disorders, whilst gene-based analysis identified 17 likely shared genes (Adewuyi *et al.*, 2022). Pathway-based analysis of overlapping genes identified multiple biological pathways involved in both diseases relating to sex hormones and physiology (Adewuyi *et al.*, 2022). Therefore, there is strong evidence for a shared genetic liability to endometriosis and asthma in the absence of a causal relationship (Adewuyi *et al.*, 2022). The significant genetic correlation, and shared causal variants and genes suggest that asthma and endometriosis are likely linked by shared molecular pathways (horizontal pleiotropy), rather than a causal effect of one disease on the other (Adewuyi *et al.*, 2022). Based on these findings, the authors recommend concurrent screening for both conditions, as well as the consideration of treatment options that target these shared pathways (Adewuyi *et al.*, 2022). Asthma may also be a useful disease-predictive factor for endometriosis, given it often onsets early in life.

### Gastrointestinal traits and endometriosis

Gastrointestinal disorders have been strongly implicated in endometriosis. Gastrointestinal complaints within the last year were reported by 85% of an endometriosis cohort (Ek *et al.*, 2015). Irritable bowel syndrome shares the symptoms of abdominal pain and chronic inflammation with endometriosis and has increased prevalence in women with endometriosis (OR = 3.26, 95% CI = 1.97–5.39) (Chiaffarino *et al.*, 2021). The genetic relationship of gastritis/duodenitis and gastro-oesophageal reflux disease (GORD) has been assessed by Adewuyi *et al.* (2021). Interestingly, this was motivated through identifying pathways associated with gastric mucosa abnormality as associated with endometriosis and depression (Adewuyi *et al.*, 2021). A significant genetic correlation is present between endometriosis and GORD (*r*_g_ = 0.24) and endometriosis and gastritis/duodenitis (*r*_g_ = 0.18) (Fig. 3) (Adewuyi *et al.*, 2021). A causal relationship of GORD on endometriosis was found via the IVW method (OR = 1.30), with no evidence for heterogeneity or directional pleiotropy (Fig. 4) (Adewuyi *et al.*, 2021). This relationship was supported by MR-PRESSO, but not by the WM model or the MR-Egger model, and leave-one-out analysis revealed the association was not driven by individual SNPs (Adewuyi *et al.*, 2021). The reverse direction, endometriosis on GORD, was not significant, however the heterogeneity tests had small *P* values (IVW Cochran’s *Q* statistic *P *=* *1.52 × 10^−8^) (Adewuyi *et al.*, 2021). There was no evidence of genetic liability to endometriosis causing gastritis/duodenitis, whilst the absence of genome-wide significant SNPs for gastritis/duodenitis prevented assessment of its causality of endometriosis (Adewuyi *et al.*, 2021). Despite the identification of a strong genetic relationship between the gastric traits, endometriosis and depression (Adewuyi *et al.*, 2021), estimation of each pairwise genetic correlation and MR result, independent of the genetic effects of the remaining trait, was not considered. Nevertheless, consideration of the comorbidity of endometriosis and gastric traits is important for managing the symptoms of both diseases. Given the identified causative role of GORD on endometriosis, treatment of GORD may assist in the management of endometriosis (Adewuyi *et al.*, 2021). This concept is supported by epidemiological evidence for an influence of diet on endometriosis and gastric symptoms (Adewuyi *et al.*, 2021). Further, non-steroidal anti-inflammatory drugs are often prescribed for endometriosis-associated pain; however, their appropriateness should be considered as they have a side effect of gastrointestinal injury (Adewuyi *et al.*, 2021).

### Psychological traits and endometriosis

A link between endometriosis and psychological traits may be expected due to the substantial pain some individuals with endometriosis experience, however genetic evidence suggests the relationship may be more complex. Epidemiological studies have found anxiety (HR = 1.38–1.44) and depression (HR = 1.48–1.56) are often seen in endometriosis cases (Chen *et al.*, 2016; Estes *et al.*, 2021), along with self-directed violence (HR = 2.03) (Estes *et al.*, 2021). Characterization of the link using genetic data has been performed for depression. Endometriosis is genetically correlated with depression (*r*_g_ = 0.27, Fig. 3) (Adewuyi *et al.*, 2021). A shared genetic architecture was additionally supported by a cross-disorder meta-analysis that identified shared 20 genomic loci and a gene-based analysis which identified 22 genes shared between disorders (Adewuyi *et al.*, 2021). However, few of these genes replicated with genome-wide significance in an independent sample (Adewuyi *et al.*, 2021). MR analyses indicated a causal effect of genetic liability to depression on endometriosis (OR = 1.26) via the IVW model, which is supported by the WM model (OR = 1.24), but not the MR-Egger model (Fig. 4) (Adewuyi *et al.*, 2021). There was evidence for significant heterogeneity, yet use of MR-PRESSO to remove outlier SNPs supported the causal relationship (Adewuyi *et al.*, 2021). Leave-one-out MR analysis indicated the result could not be attributed to individual SNPs, nor could the relationship be explained by reverse causality (Adewuyi *et al.*, 2021). The causative effect of depression on endometriosis was replicated in independent GWAS datasets, further strengthening the proposed relationship. The biological mechanism for this causal relationship is unclear, however the authors hypothesize immune-related pathways may explain the link (Adewuyi *et al.*, 2021). Arguably, the most interesting message from this study was that endometriosis-associated pain is not the sole factor linking endometriosis with depression, rather, there is a biological basis for their comorbidity (Adewuyi *et al.*, 2021). Knowledge of the relationship between endometriosis and depression is not only important for understanding the biology and pathogenesis of both diseases but also can be applied to disease prediction and monitoring as well as holistic patient care, ensuring appropriate resources are available to monitor and support the mental health of women with endometriosis.

### Anthropometric traits and endometriosis

The relationship between endometriosis and anthropometric traits has been extensively debated. A meta-analysis of the epidemiological studies investigating the relationship between endometriosis and BMI indicated that higher BMI may be associated with a decreased risk for endometriosis (Liu and Zhang, 2017). Interestingly, women with BMIs in the obese range have higher revised American Fertility Society scores (an endometriosis severity scoring system) compared to women with normal and pre-obese BMIs (Holdsworth-Carson *et al.*, 2018). Another meta-analysis reports being underweight as a risk factor for endometriosis, whilst being overweight or obese had no effect (Jenabi *et al.*, 2019). Measured height and genetically predicted height was not associated with endometriosis in a Han Chinese population (Chiou *et al.*, 2022). Therefore, further investigation into the link is needed. Two studies have assessed the causal nature of several anthropometric traits in European females with endometriosis: Garitazelaia *et al.* (2021) and Venkatesh *et al.* (2022), both using female-specific GWAS summary statistics. Garitazelaia *et al.* (2021) found a preventative effect of increased weight and BMI on endometriosis, but not for height or waist-to-hip ratio (WHR). However, these significant findings were not consistent across sensitivity analyses, although all three models showed concordant directionality of effects (Garitazelaia *et al.*, 2021). Furthermore, the assumptions of MR were not tested, and the endometriosis dataset had limited power, being generated from only 3380 endometriosis cases (Garitazelaia *et al.*, 2021). Venkatesh *et al.* (2022), using better-powered GWAS summary statistics for endometriosis (12 210 cases), found a causative effect of WHR and WHR adjusted by BMI (WHRadjBMI), but not BMI, on endometriosis (WHR and WHRadjBMI OR_IVW_ = 1.24). MR-Egger’s intercept indicated there was no horizontal pleiotropy, although there was significant heterogeneity indicated by Cochran’s *Q* statistic (Venkatesh *et al.*, 2022). Further, adjustment for leptin and insulin attenuated the causal estimate of WHR and WHRadjBMI on endometriosis, rendering it non-significant (Venkatesh *et al.*, 2022). The authors also assessed the causation of endometriosis on WHR, WHRadjBMI and BMI, and noted no significant causal estimates, although heterogeneity was present (Venkatesh *et al.*, 2022). Whilst these studies have focused on European individuals, Masuda *et al.* (2020) assessed the effect of BMI on endometriosis in a Japanese population. IVW, MR-Egger, and MR-PRESSO methods were not significant, nor was there evidence of directional pleiotropy (MR-Egger intercept not significant) or the estimate being driven by an individual SNP in leave-one-out sensitivity analysis (MaSuda *et al.*, 2020). However, this analysis was likely underpowered as the GWAS summary statistics used for endometriosis were generated from 645 cases (MaSuda *et al.*, 2020).

Another study by Rahmioglu *et al.* (2015) assessed the SNP-level overlap between endometriosis and BMI and WHRadjBMI, which also used a small set of endometriosis summary statistics (3194 cases and 7060 controls). Considering loci with genome-wide significance for each trait, two endometriosis loci have lower *P* values than expected by chance in the WHRadjBMI GWAS: intergenic 7p15.2 and *WNT4*, and two of the 16 WHRadjBMI loci had *P *<* *0.01 in the endometriosis GWAS: intergenic 7p15.2 and *GRB14* (Rahmioglu *et al.*, 2015). A statistically significant enrichment was seen with variants associated with endometriosis at *P *<* *1 × 10^−3^ and variants associated with WHRadjBMI at *P *<* *0.05, and vice versa, but not with BMI (Rahmioglu *et al.*, 2015). This relationship was strengthened when restricting to stage B endometriosis cases (Rahmioglu *et al.*, 2015). Endometriosis cases and controls did not clearly differ in polygenic risk score for WHRadjBMI nor BMI, suggesting that the overlap in risk loci does not have concordant genome-wide directional effects (Rahmioglu *et al.*, 2015). This inconsistent directional effect may explain the variable MR results. Indeed, intergenic 7p15.2 and *WNT4* risk loci show opposite directions of effect for WHRadjBMI and endometriosis, whilst the directionality is consistent for the *GRB14* SNP (Rahmioglu *et al.*, 2015). Annotation of the shared loci further highlighted SNPs near *KIFAP3*, and *CAB39L*, suggesting potential pleiotropic functions for these genes (Rahmioglu *et al.*, 2015)*.* The authors concluded that the genetic basis of endometriosis and fat distribution is overlapping, and the unclear epidemiological results can be attributed to the lack of a consistent directional effect of overlapping variants (Rahmioglu *et al.*, 2015). Together, these results suggest that the epidemiological evidence for an association between endometriosis and obesity-related traits is most likely explained by overlapping risk variants rather than a causal link. Lifestyle changes initiated by the burden of endometriosis (e.g. pain) rather than the disease itself may also be responsible.

The assessment of the causality of obesity-related traits with endometriosis is limited by the life stage at which the measurement was taken, in that weight is subject to change across an individual’s lifespan, in response to factors such as age, lifestyle, and the impact of disease. Two studies have assessed the causative effect of early weight on endometriosis. He *et al.* (2022) found no evidence for a causative effect of birthweight on endometriosis using multiple MR methods, contradicting most epidemiological evidence (Olšarová and Mishra, 2020). Yan *et al.* (2022) investigated the effects of childhood BMI on endometriosis. A significant, although small negative causative effect was found by the IVW method (OR = 0.995), but this was not supported by the WM method, nor MR-PRESSO analysis (Yan *et al.*, 2022). Further, one SNP (rs12041852) showed evidence of driving the association in leave-one-out sensitivity testing (Yan *et al.*, 2022). Therefore, due to heterogeneity, and the timely measurement of weight traits, a clear influence of obesity-related traits on or by endometriosis cannot be ascertained.

### Insights into shared candidate target genes and pathways

**Figure 5.**
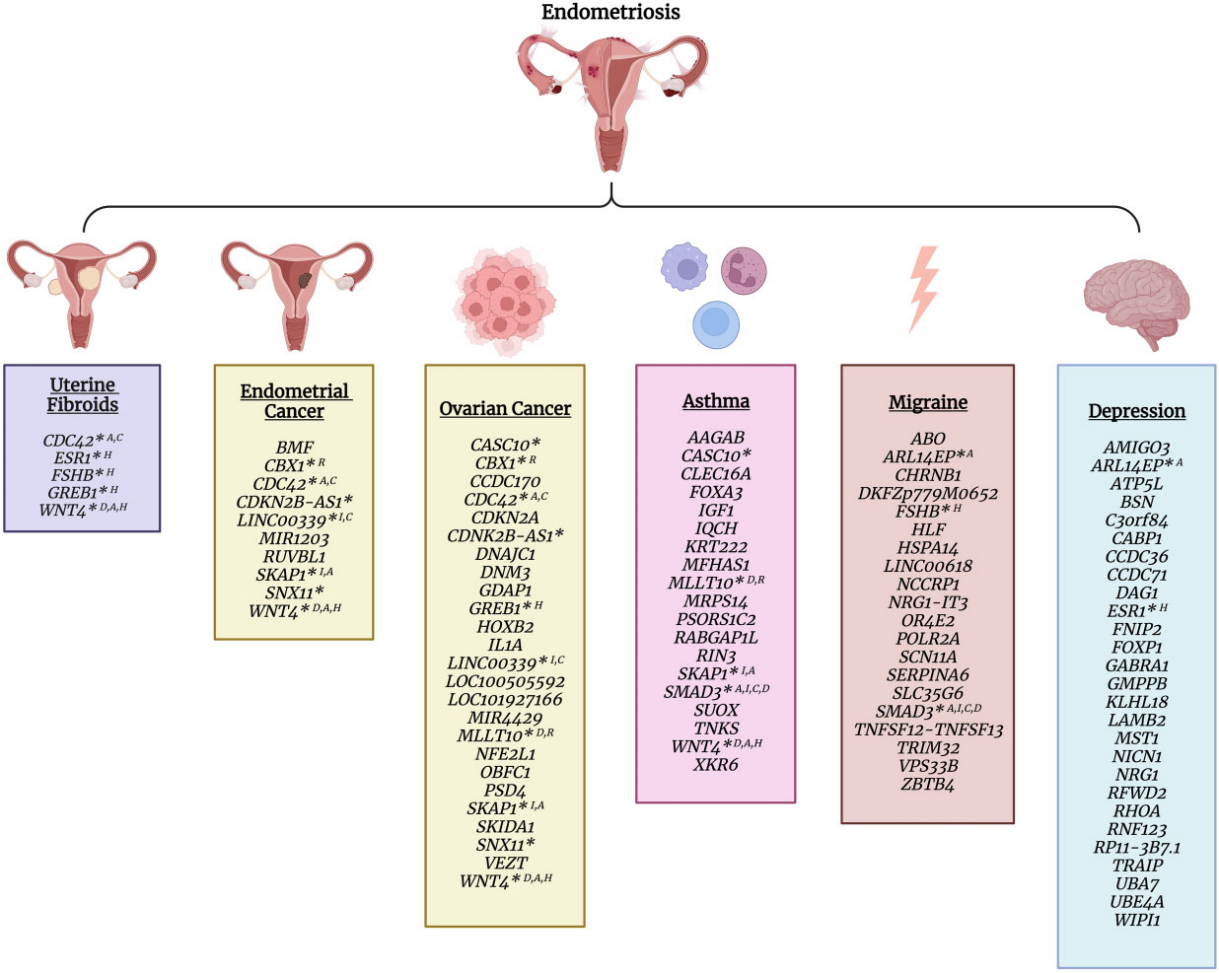
**Candidate target genes shared between endometriosis and comorbid traits.** Target genes shared between endometriosis and uterine fibroids (Gallagher *et al.*, 2019), endometrial cancer (Kho *et al.*, 2021), epithelial ovarian cancer histotypes (Rueda-Martinez *et al.*, 2021; Mortlock *et al.*, 2022), asthma (Adewuyi *et al.*, 2022), migraine (Adewuyi *et al.*, 2020), and depression (Adewuyi *et al.*, 2021). * indicates genes shared between endometriosis and multiple other traits; A, C, H, I, D, and R indicate the gene has been associated with adhesion, cell cycle regulation, hormonal, immune, cell differentiation and DNA repair pathways, respectively. Created with BioRender.com.

Analyses conducted by the aforementioned studies highlighted several potential target genes implicated in endometriosis and its comorbidities (Fig. 5). Not only are candidate target genes shared between endometriosis and individual comorbidities, but many are common across multiple comorbid traits and are enriched in similar biological pathways. Genes shared between endometriosis, uterine fibroids, ovarian and endometrial cancer, asthma and migraine (*CDC42*, *WNT4*, *SMAD3*, *SKAP1*) were enriched in cell adhesion pathways (Gallagher *et al.*, 2019; Adewuyi *et al.*, 2020, 2022; Kho *et al.*, 2021; Mortlock *et al.*, 2022). Similarly, genes shared between endometriosis, uterine fibroids, ovarian cancer, migraine and depression (*RHOA*, *FOXP1*, *ESR1*, *WNT4*, *GREB1*, *FSHB*), and endometriosis, migraine and asthma (*IGF1*, *SMAD3*, *MFHAS1*), were enriched in hormone receptor signalling and inflammation pathways, respectively (Adewuyi *et al.*, 2020, 2021, 2022; Mortlock *et al.*, 2022).

Individual candidate target genes identified between multiple endometriosis comorbidities include genes in the 1p36.12 locus, *ESR1*, *GREB1*, *FSHB*, *SKAP1*, *CASC10*, *MLLT10*, *ARL14EP*, *SNX11*, *CBX1*, *CDKN2B-AS1*, and *SMAD3* (Fig. 5). Genes in the 1p36.12 locus (*CDC42*, *WNT4*, and *LINC00339*) have been associated with endometriosis, uterine fibroids, ovarian and endometrial cancers, and asthma (Gallagher *et al.*, 2019; Kho *et al.*, 2021; Rueda-Martinez *et al.*, 2021; Adewuyi *et al.*, 2022; Mortlock *et al.*, 2022). Cell division cycle 42 (*CDC42*) has been shown to regulate cell cycle, migration, invasion and adhesion and has been associated with cancer progression (Qadir *et al.*, 2015). WNT family member 4 (*WNT4*) activates WNT/β-catenin signalling and plays an important role in the development and differentiation of many cell types during embryonic development and adult homeostasis, including the female reproductive system (Zhang *et al.*, 2021). *WNT4* also has a pro-carcinogenic role in many cancer types and is involved in immune and respiratory physiology pathways (Zhang *et al.*, 2021; Adewuyi *et al.*, 2022). *LINC00339* is a long non-coding, and competing endogenous RNA, affecting RNA metabolism, and modulating downstream target genes. It has been shown to promote tumour progression and invasion and affects vital pathways, including the WNT/β-catenin pathway, MAPK signalling pathways, and RhoA pathway (Wu *et al.*, 2022). *LINC00339* expression in endometrial stromal cell lines has also demonstrated a role in immune homeostasis mechanisms (Holdsworth-Carson *et al.*, 2021). Similarly, *SKAP1* and *MLLT10*, candidate genes for endometriosis, ovarian cancer and asthma, have also been linked to adhesion and immune regulation and hematopoietic differentiation. Src kinase associated phosphoprotein 1 (*SKAP1*) encodes an immune cell adapter protein that regulates T-cell receptor signalling and adhesion (Wang and Rudd, 2008). Mix-lineage leukaemia translocated to 10 (*MLLT10*) is a transcription factor that is involved in several chromosomal rearrangements resulting in leukaemias. It has been linked to regulation of chromatin structure and DNA damage response and is believed to be important in early development and maintenance and differentiation of hematopoietic stem cells (Deutsch and Heath, 2020). Based on their known functions, all three genes at the 1p36.12 locus have the potential to contribute to the formation and progression of lesions characteristic of endometriosis, fibroids and cancer whilst *WNT4*, *LINC0039*, *MLLT10*, and *SKAP1* have known roles in respiratory physiology and/or immune regulation, which are also important factors in asthma and cancer.

Several hormone-related genes were also identified as candidate target genes for endometriosis and its comorbidities. Oestrogen Receptor 1 (*ESR1*), a transcription factor important for hormone binding (Klinge, 2001), was associated with endometriosis, uterine fibroids and depression (Gallagher *et al.*, 2019; Adewuyi *et al.*, 2021). Previous studies also link the gene to breast cancer (Dunning *et al.*, 2016; Michailidou *et al.*, 2017). Growth regulating oestrogen receptor binding 1 (*GREB1*), an early response gene in the ER-regulated pathway (Hodgkinson *et al.*, 2018), was associated with endometriosis, uterine fibroids and ovarian cancer. FSH subunit beta (*FSHB)*, encoding the beta subunit of a pituitary glycoprotein that indices gamete production, was associated with endometriosis, uterine fibroids and migraine (Gallagher *et al.*, 2019; Adewuyi *et al.*, 2020), and previous studies also link it to reproductive lifespan, menstrual cycle characteristics, FSH concentrations, and PCOS (McGrath *et al.*, 2021). Association of these genes with endometriosis and its comorbidities suggests that dysregulation of hormone signalling may predispose women to a range of conditions spanning gynaecological, psychological, and pain disorders.

Functional evidence, including chromatin interactions and expression quantitative trait loci (Kho *et al.*, 2021; Mortlock *et al.*, 2022), suggests regulation of candidate target genes may be tissue and cell-type specific and therefore further studies are needed to determine the role of these genes in the pathogenesis of each of these diseases. Collectively, shared target genes and pathways suggests that dysregulation of cell adhesion, immune regulation and hormone signalling may be important pathological mechanisms underlying endometriosis and several comorbid traits, and genetic variants influencing these mechanisms may predispose women to multiple diseases.

## Discussion

Many studies have attempted to characterize the relationship between endometriosis and its comorbid conditions using genomic data with the goal of improving our understanding of the disease. The interpretation of the outcomes of these studies is dependent on the assumptions of the performed analyses. There is evidence for a genetic correlation between endometriosis and uterine fibroids (Gallagher *et al.*, 2019), clear cell, endometrioid and high grade serous ovarian cancer (Mortlock *et al.*, 2022), melanoma in females (Yang *et al.*, 2021), GORD, gastritis/duodenitis, depression (Adewuyi *et al.*, 2021), asthma (Adewuyi *et al.*, 2022), and migraine (Adewuyi *et al.*, 2020) (Fig. 3), suggesting genetic predisposition to endometriosis has the potential to affect many biological systems. There is robust evidence for a causal relationship of genetic liability of depression on endometriosis (Adewuyi *et al.*, 2021), and endometriosis on ovarian cancer subtypes (Mortlock *et al.*, 2022) and uterine fibroids (Gallagher *et al.*, 2019). Whilst other significant MR results have been reported, the assumptions of MR were not comprehensively tested or were demonstrated to be violated. Furthermore, significant results may be biased by unknown confounders and non-significant MR results are not definitive as they may be updated with the availability of greater powered or female-specific GWAS summary statistics.

Beyond the assumptions concerning the statistical methodology, there are other challenges and biases in studying the crossover between endometriosis and its comorbidities. Firstly, thus far most studies have not considered the variability in disease presentation of endometriosis. Individuals with endometriosis can have lesions located in multiple locations, and varying severities and types of pain, and they may or may not have infertility. Epidemiological studies specific to patients with specific lesion locations, or symptom presence, have not been conducted for any trait except for ovarian cancer, where it is known that the increased risk is limited to those with the endometrioma lesion location. It has been suggested that endometrioma, superficial peritoneal lesions and deep infiltrating lesions be considered as distinct entities (Nisolle and Donnez, 1997), yet such clinical data is rarely available alongside matched genetic data. Additionally, given the invasive nature of diagnosis, individuals with severe pelvic pain or with infertility are more likely to seek a diagnosis, as opposed to those with milder symptoms. Therefore, there is inherent bias in endometriosis case datasets to ascertain those with more severe symptoms. Another factor that impedes cross trait analysis by causing spurious genetic correlations is misdiagnosis (Gratten and Visscher, 2016). Endometriosis shares symptoms with many other diseases, so it is likely that endometriosis can be mistaken for another disease, therefore amplifying the correlation between the two diseases. Unless a trait is sex-specific, most GWAS studies are performed on a combined male-female dataset. Consequently, in the studies described here, combined GWAS summary statistics are generally used in such scenarios with the exception of a few studies using female-specific summary statistics for non-female-specific traits (Garitazelaia *et al.*, 2021; Yang *et al.*, 2021; Venkatesh *et al.*, 2022). In the instance of melanoma, the genetic correlation with endometriosis was significant in a female-specific melanoma dataset, but did not reach significance in a combined or male-specific melanoma dataset (Yang *et al.*, 2021). Therefore, it is recommended that genetic comorbidity analyses are carried out in female-only datasets, where possible. An oversight by many studies is the inclusion of adenomyosis cases in the endometriosis case cohort. International Classification of Diseases (ICD)10 diagnostic codes are used in many large biobanks and healthcare registries. The ICD10 diagnostic code N80 for Endometriosis includes nine subcodes, one of which is ‘N80.0 Endometriosis of the Uterus’, i.e. adenomyosis. Whilst endometriosis and adenomyosis share many features, they are currently considered distinct diseases. The availability and accessibility of GWAS statistics generated in published studies can also limit the ability of researchers to include well powered datasets in their analyses. Requirements by leading journals to make datasets publicly available will alleviate this challenge in future. Another challenge in use of summary statistics from GWASs is that the X chromosome is often omitted, despite being included on the genotyping chip (Wise *et al.*, 2013). Further, even if the X chromosome is present in the GWAS summary statistics, many analysis software programs omit it (e.g. LDSC, commonly used for genetic correlation; Bulik-Sullivan *et al.*, 2015a,b). Given the X chromosome contains ∼5% of the genes in the human genome it is likely to provide biological insights. Lastly, a widespread issue in genomic research is the poor availability of non-European data. Most genetic results in this review have been performed in datasets of predominantly European ancestry, which limits the cross-ancestry translation of these findings.

The genetic evidence supports the epidemiological data suggesting that individuals with endometriosis are at an increased risk of a diverse range of other conditions. Characterization of the genetic overlap with a number of these conditions has highlighted genes and pathways involved in both diseases and has emphasized the heterogeneity of the disease. However, the assessment of causal relationships with MR has been limited by the presence of possible confounders and difficulties resolving potential pleiotropic pathways. Well-designed and well-powered MR analyses could validate pleiotropic and potential causal relationships between endometriosis and its comorbidities, identifying risk factors and pathogenic pathways that can improve prognostic counselling for patients with endometriosis. The identification of risk factors is particularly important given the lengthy diagnostic delay and prevalence of unnecessary surgeries; one in four women undergoing surgery for endometriosis will not receive an endometriosis diagnosis (Kazanegra *et al.*, 2008; Stegmann *et al.*, 2008; Fernando *et al.*, 2013). Knowledge of a genetic link between migraine, uterine fibroids, asthma, ovarian cancer, GORD, gastritis, and depression with endometriosis highlights not only these traits but also their underlying genetic predispositions, as factors that could be used to develop, and/or improve, diagnostic questionnaires and tools that can support endometriosis diagnosis. Integration of large disease-relevant omic datasets will facilitate the functional annotation of shared genetic risk factors and identification of shared genes and biological pathways. Overall, the published data suggest that investigation of endometriosis using genetic overlap with its comorbid disorders provides a promising strategy to better understand pathological mechanisms and inform clinical decisions.

## Data Availability

No new data were generated or analysed in support of this research.
